# Salmonella associated endogenous endophthalmitis: a case study and review of literature

**DOI:** 10.1186/s12348-026-00632-8

**Published:** 2026-09-19

**Authors:** Karande Sayali A, Jadhav Lakdawala Sonal, Jay Sheth

**Affiliations:** 1 Shantilal Shanghvi Eye Institute, Mumbai, India; 2 Panorama Eye Clinic & Sohum Eye Care Centre, Mumbai, India

**Keywords:** Endogenous endophthalmitis, Salmonella, Paratyphi A

## Abstract

**Purpose:**

To report a rare case of bilateral endogenous endophthalmitis caused by Salmonella Paratyphi A in an immunocompetent patient and to review the relevant literature.

**Method:**

Brief report.

**Results:**

A 23-year-old woman presented with acute bilateral vision loss over one day. She had previously received systemic corticosteroids for a presumed Vogt–Koyanagi–Harada syndrome and was administered oral steroids. At presentation, examination revealed conjunctival congestion, Descemet’s folds, fibrinous anterior chamber reaction, and hypopyon in the left eye, with obscured; fundus details. B-scan ultrasonography demonstrated a thickened retinochoroidal complex with shallow choroidal detachments and anechoic vitreous cavities. Blood culture grew Salmonella Paratyphi A and vitreous cytology showed abundant monocytes. She received intravitreal vancomycin (1 mg/0.1 ml) and ceftazidime (2.25 mg/0.1 ml), IV ceftriaxone 2 g/day guided by susceptibility, topical prednisolone acetate 1% and atropine 1%, and a tapering course of oral steroids. Following resolution of anterior segment inflammation, she underwent pars plana vitrectomy with lensectomy and silicone oil endotamponade in both eyes. Postoperatively, best-corrected visual acuity improved to 20/250 (OD) and 20/200 (OS).

**Conclusion:**

While Salmonella-associated endogenous endophthalmitis is typically associated with poor visual outcomes, this bilateral Paratyphi A case demonstrates that prompt recognition and aggressive combined medical and surgical management can achieve meaningful visual recovery.

## Introduction

Endogenous endophthalmitis arises from hematogenous seeding of intraocular tissues and, though uncommon (≈ 2–8% of all endophthalmitis), represents a significant diagnostic and therapeutic challenge. It frequently occurs in patients with systemic vulnerability, such as diabetes, malignancy, immunosuppression, or extremes of age, but can occasionally affect immunocompetent individuals. [1] Fungal pathogens and gram-positive bacteria are the most common causes, [2, 3] whereas Salmonella-associated endogenous endophthalmitis (SAEE) remains exceptionally rare and typically fulminant, with poor visual prognosis. This report describes a bilateral Paratyphi A case in an immunocompetent patient along with an existing review of literature.

## Methodology

Clinical data, including presentation, investigations, management, and follow-up, were extracted from electronic medical records, and written informed consent was obtained. The study adhered to the Declaration of Helsinki. Routine microbiological testing of blood, urine, and vitreous samples was recorded. PubMed searches using “endogenous endophthalmitis,” “Salmonella endophthalmitis,” “extraintestinal manifestations,” “typhoid fever relapse,” and “typhoid” identified case reports and series; additional references were retrieved from bibliographies. Inclusion required clinical features of endogenous endophthalmitis and culture-confirmed Salmonella infection from blood or vitreous.

### Case report

A 23-year-old woman developed sudden bilateral visual loss over 24 h, with presenting acuity limited to hand movements in both eyes (OU). She had been treated elsewhere with IV methylprednisolone (1 g/day) followed by oral steroids for a presumed Vogt–Koyanagi–Harada diagnosis. On presentation, both eyes exhibited conjunctival congestion, Descemet’s folds, and a fibrinous anterior chamber membrane; a hypopyon was present in the left eye, and fundus visualization was hindered by a yellowish glow (Fig. 1A, B). Ocular motility was full and painless. B-scan ultrasonography revealed a thickened retinochoroidal complex, shallow choroidal detachments (CDs) in multiple quadrants, and anechoic vitreous (Image 1 C, D). Contrast MRI of the brain and orbit showed mild preseptal edema with CDs. Past history elicited a febrile illness three days prior; laboratory evaluation revealed thrombocytopenia.

**Fig. 1:**
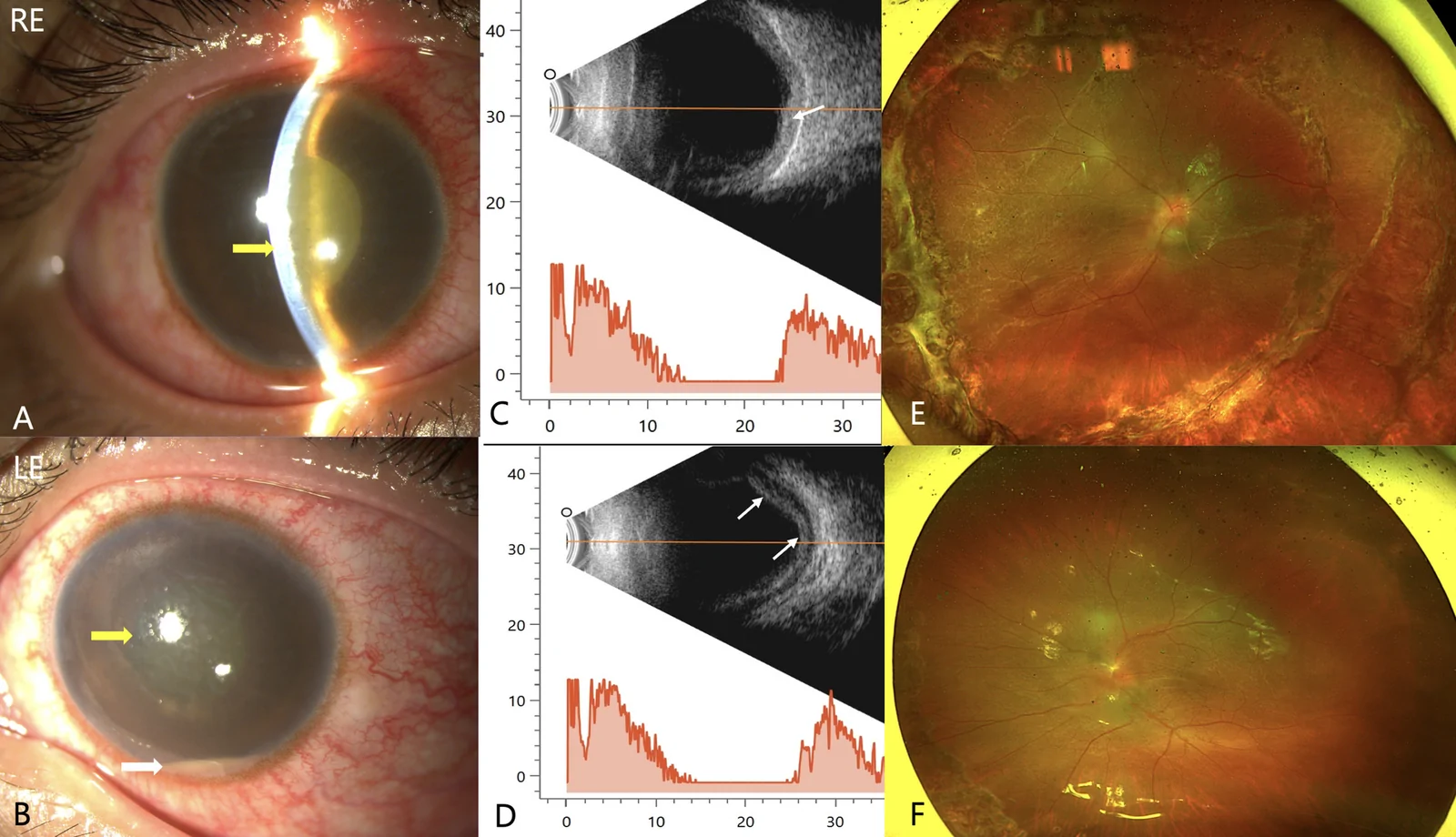
At presentation: (**A**) Slit section right eye showing conjunctival congestion, Descemet’s fold and fibrinous reaction in the anterior chamber; (**B**) Diffuse illumination left eye showing conjunctival congestion, Descemet’s fold and 1 mm hypopyon; (**C**, **D**) Ultrasound B-scan showing thickened retino-choroidal complex with shallow choroidal detachments; (**E**, **F**) Ultrawide field postoperative fundus image of both the eyes following vitrectomy showing attached retina with silicon oil endotamponade

Given suspicion for bilateral endogenous endophthalmitis, left eye vitreous biopsy was performed with intravitreal vancomycin (1 mg/0.1 ml) and ceftazidime (2.25 mg/0.1 ml), alongside blood and urine cultures. Blood culture grew abundant Salmonella Paratyphi A, and vitreous cytology showed abundant monocytes. She was initiated on IV ceftriaxone 2 g/day per susceptibility, topical prednisolone acetate 1%, atropine 1%, and a tapering course of oral steroids. Once anterior segment inflammation resolved, she underwent pars plana vitrectomy with lensectomy and silicone oil endotamponade in both eyes (Fig. 1E, F). Postoperatively, her visual acuity improved to 20/250 OD and 20/200 OS which was maintained for 6 months.

## Discussion

SAEE is uncommon but clinically important because of its fulminant course and historically poor visual outcomes. Reported causative serotypes include *S. Typhi*,* S. Typhimurium*,* S. Enteritidis*,* S. choleraesuis*, and isolated reports of *S. arizona* and *S. London* (Table 1) [4–16]. To our knowledge, *S. Paratyphi A* has not been previously reported as a cause of endogenous endophthalmitis, making this a notable addition to the spectrum of implicated serotypes.

The pathogen is transmitted via the faecal–oral route through contaminated food and water, with animals and poultry serving as reservoirs. [17, 18] Extra-intestinal manifestations are increasingly recognized. Risk factors for complications include bacterial virulence, infective dose, host immunity, prior exposure, and local tissue defenses. In typhoid fever, infection evolves through three clinical stages of roughly one week each. Early transient bacteremia allows dissemination from the intestinal mucosa to distant sites, including the eye. As bacterial load increases, sustained secondary bacteremia triggers macrophage activation and cytokine release, coinciding with symptomatic disease. Persistent bacteremia permits seeding of extra-intestinal sites, producing localized infection. [19] The vitreous, as an immune-privileged compartment, favors microbial proliferation. Once the blood–ocular barrier is breached, bacteria localize preferentially to the choroid and retina, triggering intense, rapidly destructive inflammation.

Host and pathogen factors, including immune status, comorbidities, bacterial virulence, and inoculum size, significantly influence disease severity. Prior or prolonged systemic corticosteroids may suppress early inflammatory signs and accelerate intraocular infection, as likely occurred in our patient. SAEE can present atypically; bilateral involvement and CDs, as observed here, may obscure diagnosis and delay treatment.

Early microbiological sampling is therefore essential. Blood cultures and vitreous sampling when feasible maximize diagnostic yield and enable susceptibility-guided therapy, as empiric regimens may not cover resistant strains. Intravitreal vancomycin plus ceftazidime remains appropriate initial therapy, while systemic antibiotics should be culture-directed, with third-generation cephalosporins (e.g., ceftriaxone) recommended for severe enteric infection. Geographic variations in antimicrobial resistance further emphasize the need for targeted therapy.

In fulminant SAEE, vitrectomy is often decisive. Surgery reduces microbial and inflammatory burden, removes debris, and enhances intraocular drug penetration. [1] When combined with timely, culture-directed medical therapy, prompt vitrectomy can preserve useful vision. Although many reported cases progress to no light perception or require enucleation, our patient’s recovery demonstrates that early diagnosis, rapid culture confirmation, and timely surgical intervention can achieve meaningful visual restoration, even in bilateral disease.

From a public-health perspective, SAEE highlights the ocular risks of systemic Salmonella infections in endemic regions (notably South and Southeast Asia) and underscores the importance of improving water and food safety. Clinically, this case supports three practical recommendations: (1) maintain a high index of suspicion for endogenous infection in any acute, severe intraocular inflammation, particularly following a recent febrile illness; (2) obtain blood and vitreous cultures prior to prolonged empirical immunosuppression; and (3) initiate culture-directed systemic antibiotics alongside prompt intravitreal therapy and timely vitrectomy when indicated.

This report is limited by its single-patient design, which restricts the generalisation of the observations. Although, *Salmonella Paratyphi A* was isolated from blood cultures in the setting of systemic febrile illness and bilateral ocular inflammation, direct microbiological confirmation from intraocular cultures was not available. Prior administration of systemic corticosteroids before referral may have likely modified the disease course and resulted in poor yield. Finally, the follow-up period was limited to six months, precluding assessment of long-term visual and anatomical outcomes. Nonetheless, the case expands the spectrum of Salmonella serotypes implicated in ocular infection and highlights a potentially modifiable window for intervention.

In conclusion, Salmonella species can rarely seed the eye during acute enteric infection or relapse. In endemic settings, particularly India and parts of Asia, clinicians should include SAEE in the differential for acute intraocular inflammation, obtain early cultures, avoid prolonged empirical immunosuppression, and consider aggressive combined medical and surgical therapy to optimize visual outcomes.

**Table 1:** Summary of published cases on Salmonella-associated endogenous endophthalmitis (SAEE), highlighting patient demographics, systemic associations, microbiological profile, management strategies, and visual outcomes

Case Report	Age/sex	Systemic status	Signs and Symptoms	Lab Diagnosis		Treatment	Outcome	Salmonella species
1. Corman LI et al.4 (1979)	7 week/M	Nil	OD Proptosis, Periorbital swelling, Leucocoria, Pneumonia, Diarrhoea	Aqueous culture +ve	Systemic Ampicillin		Enucleation	*S. enteritidis*
2. Shohet I et al.5 (1981)	1 year/M	Nil	OD Inflammation, Leucocoria, Diarrhoea	Blood, Vitreous and Stool culture +ve	Systemic Gentamycin, Cephalothin		No PL	*S. typhimurium*
3. Weinstein JM et al.6 (1982)	48 year/M	Chronic lymphoid leukaemia	OD Pain & Decreased vision, Diarrohea	Aqueous, Stool culture +ve	Systemic Chloramphenicol, Cotrimoxazole		Enucleated	*S. typhimurium*
4. Appel I et al.7 (1986)	1 year/F	Nil	OD Lid edema, Inflamation, Corneal edema, Diarrhoea, Septicemia	Blood, Aqueous and Stool culture +ve	Systemic Garamycin and Cephalothin,		Improved	*S. typhimurium*
5. Caravalho J Jr et al.8 (1990)	55 year/F	Rheumatoid arthritis	OS Diminution of vision, Redness	Blood and Vitreous culture +ve	Systemic Antibiotics not specified		Enucleation	*S.arizona*
6. Yu, YS et al.9 (2002)	3 months	Nil	OD Redness, Leukocoria, Mild fever, Diarrhoea	Vitreous culture +ve	Systemic Nafcillin, Cefotaxime, Metronidazole, Vitrectomy lensectomy		Not mentioned	*S.london* (Group E)
7. Yodprom R et al.10 (2007)	54 year/M	HIV	OS Acute painless loss of vision	Aqueous +ve	Intravitreal and Systemic Ceftriaxone		No PL	*S. choleraesuis*
8. Arora R et al.11 (2008)	32 year/M	Nil	Bilateral Proptosis, Loss of vision	Widal test +ve, Blood culture +ve	Vitrectomy, Systemic Ceftriaxone, Gentamycin		Phthisis of both eyes	*S. typhi*
9. Sinha et al. ^12^ (2012)	4 year/M	Nil	OS Inflammation, Exudates in AC	Vitreous culture +ve	Vitrectomy and, systemic gentamicin		Phthisis	*S. typhi*
10. Dadia et al.^13^ (2017)	28 year/M	Nil	OS corneal haze, hypopyon, vitreous exudates	Blood, vitreous culture +ve	Intravitreal antibiotics and oral ciprofloxacin		Evisceration	*S.typhi*
11. Mitra et al.^14^ (2024)	1. 7 months/F	Prematurity	OS lid oedema, AC exudates, leukocoria, fever	AC exudates+	Intravitreal antibiotics, systemic meropenem		Phthisis bulbi	*S.typhi*
	42 year/ M	Nil	OD defective vision, redness, swelling	Vitreous culture+, blood culture+	Vitrectomy, intravitreal antibiotics		Phthisis bulbi	*S. enterica*
13. Messalti et al.^15^ (2024)	85 year/ F	Multiple myeloma	OD defective vision, AC exudates	Vitreous culture+	Vitrectomy		20/32	*S. enteritidis*
14. Gajdzis M et al. ^16^ (2025)	2 month/ M	Young age	OD lid oedema, pupillary membrane, vitreous exudates	Intraocular contents culture	Systemic antibiotics		Enucleation	*NTS species*
	5 month/ M	Young age	Lid oedema, AC hypopyon, vitreous exudates	Vitreous tap, blood culture	Intravitreal and systemic antibiotics		Enucleation	*NTS species*
16. Our case	23 year/F	Nil	OU lid oedema, AC exudates, yellow fundus glow	Blood culture, Vitreous culture: Monocytes	Intravitreal and systemic antibiotics		Phthisis bulbi OU	*S. Paratyphi*

PL: Perception of light

## Data Availability

No datasets were generated or analysed during the current study.

## References

[1] Sadiq MA, Hassan M, Agarwal A, Sarwar S, Toufeeq S, Soliman MK, Hanout M, Sepah YJ, Do DV, Nguyen QD (2015) Endogenous endophthalmitis: diagnosis, management, and prognosis. J Ophthalmic Inflamm Infect 5(1):32 10.1186/s12348-015-0063-y PMC4630262 26525563

[2] Schiedler V, Scott IU, Flynn HW Jr, Davis JL, Benz MS, Miller D (2004) Culture-proven endogenous endophthalmitis: clinical features and visual acuity outcomes. Am J Ophthalmol 137(4):725–731 10.1016/j.ajo.2003.11.013 15059712

[3] Sharma S, Padhi TR, Basu S, Kar S, Roy A, Das T (2014) Endophthalmitis patients seen in a tertiary eye care centre in Odisha: a clinico-microbiological analysis. Indian J Med Res 139(1):91–98 PMC3994746 24604043

[4] Corman LI, Poirier RH, Littlefield CA, Sumaya CV (1979) Endophthalmitis due to Salmonella enteritidis. J Pediatr 95:1001–1002 10.1016/s0022-3476(79)80295-4 387934

[5] Shohet I, Davidson S, Boichis H, Rubinstein E (1983) Endogenous endophthalmitis due to Salmonella typhimurium. Ann Ophthalmol 15:321–322 6362521

[6] Weinstein JM, Elliott J, Tilford RH (1982) Metastatic endophthalmitis due to Salmonella typhimurium. Arch Ophthalmol 100:293–295 10.1001/archopht.1982.01030030295013 7039571

[7] Appel I, Landman I, Savir H, Elian E (1986) Endophthalmitis due to Salmonella typhimurium. J Pediatr Ophthalmol Strabismus 23:29–30 10.3928/0191-3913-19860101-08 3512814

[8] Caravalho J Jr, McMillan VM, Ellis RB, Betancourt A (1990) Endogenous endophthalmitis due to Salmonella arizonae and Hafnia alvei. South Med J 83:325–327 Erratum in: South Med J. 1990;83:496 10.1097/00007611-199003000-00017 2346566

[9] Yu YS, Hwang SW, Lee HJ (2002) Salmonella london endogenous endophthalmitis in a healthy infant. Pediatr Infect Dis J 21:578 10.1097/00006454-200206000-00027 12182393

[10] Yodprom R, Pathanapitoon K, Kunavisarut P, Ausayakhun S, Wattananikorn S, Rothova A (2007) Endogenous endophthalmitis due to Salmonella choleraesuis in an HIV positive patient. Ocul Immunol Inflamm 15:135–138 10.1080/09273940701244228 17558841

[11] Arora R, Das S, Chauhan D, Daraius S, Narula R, Sachdev R (2008) Bilateral endogenous panophthalmitis caused by Salmonella typhi: First case report. Orbit 27:115–117 10.1080/01676830601177497 18415871

[12] Sinha MK, Jalali S, Nalamada S (2012) Review of endogenous endophthalmitis caused by Salmonella species including delayed onset Salmonella typhi endophthalmitis. Semin Ophthalmol 27(3–4):94–98 10.3109/08820538.2012.680642 22784273

[13] Dadia SD, Modi RR, Shirwadkar S, Potdar NA, Shinde CA, Nair AG (2018) Salmonella Typhi Associated Endogenous Endophthalmitis: A Case Report and a Review of Literature. Ocul Immunol Inflamm 26(4):527–532 10.1080/09273948.2017.1306085 28453408

[14] Mitra S, Meshram B, Jalali S, Basu S (2024) Clinico-Microbiological Correlation in *Salmonella* Endophthalmitis: Case Series and Review of Literature. Semin Ophthalmol 39(4):294–229 10.1080/08820538.2024.2308802 38254268

[15] Messalti O, Kaspi M, Garcin T (2024) Salmonella Enteritidis Endogenous Endophthalmitis with Abscess of Capsular Bag. Ophthalmology 131(8):974 10.1016/j.ophtha.2023.09.015 37831028

[16] Alhejaili A, Alsaif A, Madani F, Althaqib RN, Semidey V, Babgi R, Alsulaiman HM (2025) Salmonella-associated endophthalmitis in healthy infants: Two case reports and a review of the literature. Am J Ophthalmol Case Rep 40:102435 10.1016/j.ajoc.2025.102435 PMC12512158 41079014

[17] Balasubramanian R, Im J, Lee JS, Jeon HJ, Mogeni OD, Kim JH, Rakotozandrindrainy R, Baker S, Marks F (2019) The global burden and epidemiology of invasive non-typhoidal Salmonella infections. Hum Vaccin Immunother 15(6):1421–1426 10.1080/21645515.2018.1504717 PMC6663144 30081708

[18] Foodborne pathogens, Bintsis T (2017) AIMS Microbiol 3:529–563 10.3934/microbiol.2017.3.529 PMC6604998 31294175

[19] Huang DB, DuPont HL (2005) Problem pathogens: extra-intestinal complications of Salmonella enterica serotype Typhi infection. Lancet Infect Dis 5(6):341–348 10.1016/S1473-3099(05)70138-9 15919620

